# Association between C-Reactive protein and periodontitis in an obese population from the NHANES 2009–2010

**DOI:** 10.1186/s12903-023-03189-3

**Published:** 2023-07-22

**Authors:** Jiangling Sun, Wang Wang, Dongdong Li, Jukun Song, Zhu Chen, Liming Chen, Ralf Smeets, Thomas Beikler, Jan Strenge, Zhe Yang, Reinhard E. Friedrich

**Affiliations:** 1 Department of Science and Education, Guiyang Stomatological Hospital, 550002 Guizhou, China; 2 Department of Oral and Maxillofacial Surgery, University Medical Center Hamburg-Eppendorf, 20246 Hamburg, Germany; 3 Department of Periodontics, Preventive and Restorative Dentistry, University Medical Center, Hamburg-Eppendorf, 20246 Hamburg, Germany; 4 Department of Clinical Teaching, Guizhou Medical University, Guizhou Province, China; 5 Department of Oral and Maxillofacial Surgery, the Affiliated Stomatological Hospital of Guizhou Medical University, Guizhou, China; 6 Department of Periodontics, Guiyang Stomatological Hospital, Guizhou Province, China; 7 Department of Oral and Maxillofacial Surgery, Division of Regenerative Orofacial Medicine, University Medical Center Hamburg-Eppendorf, 20246 Hamburg, Germany; 8 Department of Histology and Embryology, School of Basic Medicine, Guizhou Medical University, Guiyang, Guizhou Province China

**Keywords:** Periodontitis, Obesity, C-reactive protein, Body mass index

## Abstract

**Background:**

Various data have been obtained on the relationship between body mass index (BMI) and C-reactive protein (CRP) and periodontitis. The aim of this study was to determine whether CRP/BMI are associated with periodontitis using data from the National Health and Nutrition Examination Survey (NHANES) database.

**Methods:**

A cross-sectional analysis of data from 3602 participants in the 2009–2010 NHANES cycle was performed. The definition of periodontitis was used to divide participants into four groups according to the criteria of Eke. Correlations between CRP/BMI and periodontitis were tested for statistical significance by means of descriptive statistics, multivariate regression, and subgroup-stratified analyses, with and without adjustments for confounders (such as age and sex).

**Results:**

There were no statistically significant differences (*p* > 0.05) regarding BMI and the development of periodontitis. After adjustment for age, sex, race, marital status, annual family income, alcohol consumption, hypertension, smoking, chronic pulmonary disease, cardiovascular disease, diabetes, flossing, and arthritis, CRP correlated significantly with the development of periodontitis in the subgroups stratified by obesity, with an odds ratio (OR) of 1.2 (95% CI, 1.0 to 1.5).

**Conclusion:**

Through data analysis, we found an association between CRP levels and periodontitis prevalence in the American population, although this association was only present in the obese population. While there are several hypotheses about the underlying mechanism, further studies are needed to validate these findings.

## Introduction

Periodontitis is a chronic infectious-inflammatory disease caused by plaque biofilm that is formed by bacteria deposited on tooth surfaces [1], resulting in intraoral dysbiosis and dysregulated host responses, which promote tissue damage and inhibit effective bacterial clearance [2]. Periodontitis often leads to increased bleeding on probing scores, progressive clinical attachment loss and, if left untreated, tooth loss, and is one of the sixth most prevalent diseases in humans, with an overall prevalence of 45–50% [3, 4]. Severe periodontitis affects 11.2% of the world’s population [4]. The 2012 Centers for Disease Control/American Academy of Periodontology (CDC/AAP) classification criteria presented by Eke and the 2018 European Federation of Periodontology/American Academy of Periodontology (EFP/AAP) classification criteria presented by Tonetti et al. are currently used to classify periodontitis [5, 6]. Based on the 2012 classification of periodontal disease, the 2018 criteria was upgraded for periodontitis classification and includes 4 stages and 3 grades. In this classification, clinical attachment loss (CAL), radiographic bone loss (RBL), and missing teeth due to disease are the main factors used for staging. In addition, factors such as biofilm and smoking are also included in the grading scale [6]. The 2018 periodontitis criteria showed significant agreement with the 2012 criteria in the sample of periodontitis patients included in the Brazilian Rural Intentional periodontitis survey [7].

A body mass index (BMI) of over 30 kg per square height in metres is used to define obesity [8], which has spread globally, even to low- and middle-income nations, and has been called an “epidemic” by the WHO. Over 13% of adults worldwide were obese in 2016, affecting 11% of males and 15% of females, and the incidence of obesity has nearly tripled since 1975 [9]. As the prevalence of obesity increases, there is growing concern about its negative effects on dental health, a vital component for sustaining overall well-being and quality of life [3]. Several clinical studies have shown that obesity appears to be a high-risk factor for periodontitis. A 27-year clinical study by Gorman et al. found that men with obesity had more significant clinical attachment loss and alveolar bone loss and increased probing depth [10]. However, a 4-year study of Finnish people conducted by Saxlin et al. did not find a correlation between obesity and the incidence of periodontitis [11]. According to a study conducted in Copenhagen, clinical BMI and attachment loss (AL) may be inversely related [12]. In consideration of the above controversy, more research is needed, particularly to determine whether an increased BMI is associated with periodontitis.

To screen people at high risk for periodontitis, certain inflammatory markers associated with periodontitis are considered reference indicators, such as serum C-reactive protein (CRP) [13], interleukin-6 and tumour necrosis factor levels [11, 14]. A correlation between C-reactive protein levels and periodontal disease was found in one cross-sectional study of the U.S. population older than two months using data from 1988 to 1994 [15]. This study was therefore interested in examining the potential relationship between CRP and periodontal disease in updated National Health and Nutrition Examination Survey (NHANES) data from 2009 to 2010.

Therefore, the aim of this study was to evaluate the potential association between CRP/BMI (exposure) and the development of periodontitis by using NHANES data (2009–2010).

## Methods

### Population and study design

This cross-sectional study analysed data from the NHANES, a national cross-sectional survey conducted by the Centers for Disease Control and Prevention (CDC) and published in 2013 [16]. We initially analysed periodontal data from 2009 to 2014 for 30,468 participants. The lack of CRP data from 2011 to 2014 left 10,537 participants from 2009 to 2010 with data available for analysis. This was the most recent NHANES cycle to measure blood CRP levels and conduct an updated full-mouth periodontal assessment in the USA. The inclusion criteria were as follows: (1) NHANES subjects above 30 years of age; (2) Participants whose CRP/BMI levels were measured in the NHANES; and (3) Participants in the oral health evaluation phase of the NHANES who underwent a periodontal examination. The exclusion criterion was as follows: Participants who did not undergo a thorough periodontal examination. This method removed any participants with missing data, yielding a final sample of 3,602.

### Periodontitis classification

The number of teeth, periodontal pockets, gum recession, and bleeding on probing (BOP) are all aspects of oral health. A trained professional examined recession, attachment loss, and BOP at mobile screening facilities. The NHANES 2000 Oral Health Training Manual includes thorough instructions and data. Six sites per tooth, for up to 28 teeth, were measured as part of the NHANES 2009–2010 Oral Health - Periodontal Exam. Two included measures of periodontal health were AL and probing depth (PD).

This study utilized the 2012 revision of the periodontitis classification criteria presented by Eke et al. [5]. Mild periodontitis was characterized by the presence of at least two interproximal sites with AL ≥ 3 mm or more and at least two interproximal sites with PD ≥ 4 mm (not on the same tooth) or one site with PD ≥ 5 mm. Moderate periodontitis was characterized by the presence of at least two interproximal sites with AL ≥ 4 mm (not on the same tooth) or at least two interproximal sites with PD ≥ 5 mm (not on the same tooth). Severe periodontitis was characterized by the presence of at least two interproximal sites with AL ≥ 6 mm (not on the same tooth) and at least one interproximal site with PD ≥ 5 mm. No periodontitis was defined as the absence of evidence of mild, moderate, or severe periodontitis.

### BMI classification

The heights and weights of all individuals were converted into metric measurements to the nearest inch and pound, and then BMI was calculated using a computer. Following WHO standards, the participants were included in one of three categories: underweight to normal weight (< 25 kg/m^2^), overweight (≥ 25, < 30 kg/m^2^), or obese (≥ 30 kg/m^2^) [9].

### CRP definition

CRP served as the study’s main exposure. Blood samples from both males and females over 3 years old were prepared, kept, and delivered to the University of Washington in Seattle, Washington [17]. This method utilized latex-enhanced nephelometry to quantify CRP. Particle-enhanced tests were developed based on the interaction between a soluble analyte and the corresponding antigen or antibody bound to polystyrene particles. Anti-CRP antibodies were measured by covalently attaching them to particles with a polystyrene core and hydrophilic shell. The latex particles were coated with mouse monoclonal anti-CRP antibodies and added to a diluted test sample solution. When combined with the latex particles, CRP from the test sample formed an antibody-antigen complex. A serum hypersensitive CRP threshold of 0.02 mg/ml was used. When the outcome was less than the detection threshold, the variable’s value was equal to the detection threshold divided by the square root of two [18].

### Covariates

Age in years was utilized as a continuous variable. Sex was designated as male and female. In the NHANES, race is a computed variable that is used to categorize Mexican Americans, non-Hispanic whites, non-Hispanic blacks, other Hispanics, and Other Races - Including Multi-Racial. Marital status was classified as currently married, formerly divorced, and others (widowed, living with a partner, separated). Annual family income level was classified as below $20,000, $20,000 to $55,000, or over $55,000 [19]. Alcohol consumption was classified as follows: [1] current heavy alcohol consumption (3 drinks per day for women and 4 drinks per day for men, or binge drinking 4 drinks on one occasion for women and 5 drinks on one occasion for men on 5 or more days per month); [2] moderate alcohol consumption (2 drinks per day for females, 3 drinks per day for males, or binge drinking at least twice a month); [3] mild alcohol consumption (less than or equal to 1 cup per day for women and less than or equal to 2 cups per day for men); [4] never (never drinks alcohol); and [5] former alcohol consumption (stopped drinking) [20]. Smoking status was classified as never, former, now. We categorized flossing into 4 groups: never for 0 days, rarely for 1–2 days, frequently for 3–5 days, and frequently for more than 6 days in the last week.

### Statistical analyses

Following the Analysis Recommendation of the National Center for Health Statistics (NCHS), we conducted a weighted analysis to obtain national representation. If possible, the mean ± standard deviation was utilized to describe continuous variables, while percentages (%) denote categorical variables. The *P* values were determined using a weighted chi-square test for categorical variables and a weighted linear regression model for continuous variables.

Employing multivariable regression, the relationship between CRP/BMI and periodontitis was analysed. In addition, stratified analyses of exposure factors and various populations (sex, smoking status, etc.) were conducted. To further investigate, we applied the following three models: unadjusted model, in which variables were not adjusted; Model I, adjusted for sex and age; and Model II, adjusted for age, sex, race, marital status, annual family income level, alcohol consumption, hypertension, smoking status, chronic pulmonary disease (COPD), cardiovascular disease, diabetes, flossing, and arthritis.

**Fig. 1:**
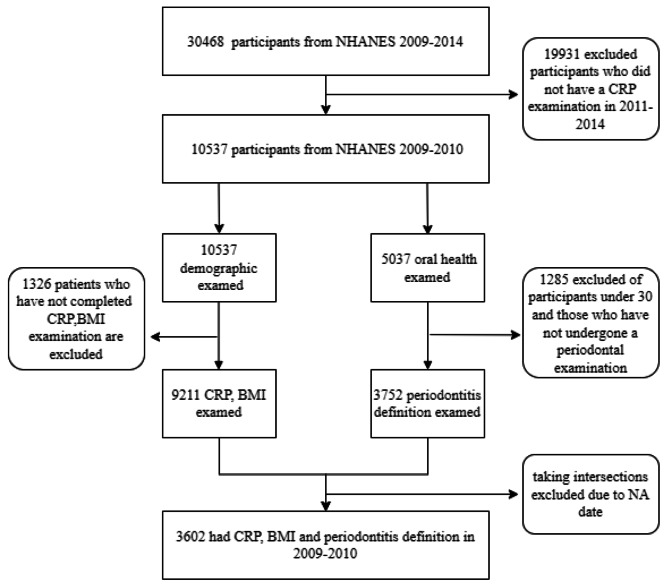
Sample selection flowchart from NHANES 2009–2010. A total of 30,468 subjects who underwent both periodontal and CRP testing and were excluded from the NA were eliminated from the trial, allowing 3602 to enter the study

The NHANES (2009–2010) database was combined with R (version 4.2.1) software to generate the results and EmpowerStats (version 4.1) was used to analyse the data. *P* < 0.05 was considered statistically significant. The flowchart in Fig. 1 shows the extensive screening process.

## Results

### Sample selection results

During the NHANES cycle that ran from 2009 to 2014, a total of 30,468 people were interviewed to obtain a representative sample of the noninstitutionalized population in the United States. Figure 1 depicts a flowchart of the sample studied. CRP levels/BMI measurements were taken and participants were evaluated for periodontitis. Of the 5037 participants who underwent oral health examinations, 3602 remained after the exclusion of those who were unable to undergo periodontal examination due to illness and those who failed to undergo the examination.

### Characteristics of participants

The demographics and population characteristics were analysed with respect to the severity of periodontal disease (no, mild, moderate, and severe disease) among the 3602 adults over 30 years old in the NHANES 2009–2010 cycle (see Table 1). Of the 3602 individuals in the analysis sample, 419 had moderate periodontitis, and 343 had severe periodontitis. There was no significant association between BMI or vitamin D consumption (*p* > 0.05) and the severity of periodontal disease. In terms of sex, the percentage of men in the severe periodontitis group was 55.9%, while that in the moderate periodontitis group was as high as 74.5%. Moreover, in the moderate group, the percentage of people who never flossed was as high as 50.6%, which was much higher than the other three categories (22.9 − 32.8%). In the severe periodontitis group, the proportion of people with a BMI ≥ 30 kg/m2 was 45.5%, higher than that in the mild to moderate groups (35.4 − 37.1%).

**Table 1:** Baseline distribution according to population characteristics and periodontitis status from the NHANES 2009-2010 cycle

	No periodontitis	Mild periodontitis	Moderate periodontitis	Severe periodontitis	*p* value
N	1580	1260	419	343	
CRP (mg/dL)	0.4 ± 0.6	0.4 ± 0.8	0.5 ± 1.0	0.4 ± 0.8	0.0163
Age (years)	47.3 ± 13.3	58.1 ± 14.0	56.6 ± 11.9	47.4 ± 13.0	< 0.0001
BMI (kg/m^2^)	29.3 ± 6.5	29.5 ± 6.7	29.0 ± 6.0	30.6 ± 6.7	0.0606
Vitamin D intake (mcg)	5.1 ± 4.3	5.1 ± 4.5	4.7 ± 3.8	5.5 ± 4.5	0.1974
Sex (%)					< 0.0001
Male	40.6	55.7	74.5	55.9	
Female	59.4	44.3	25.5	44.1	
Race (%)					< 0.0001
Non-Hispanic White	76.2	66.8	52.6	61.5	
Mexican American	5.0	9.1	16.5	13.5	
Non-Hispanic Black	8.0	12.0	16.2	14.5	
Other Hispanic	4.9	5.0	4.2	7.1	
Other Race	5.9	7.2	10.5	3.3	
Marital Status (%)					0.0020
Married	68.7	61.4	55.0	62.3	
Others	20.2	27.2	28.1	25.0	
Divorced	11.0	11.1	16.9	12.7	
Not recorded	0.0	0.3	0.0	0.0	
Annual Family Income level (%)					< 0.0001
Middle	31.8	48.8	47.8	40.5	
High	59.7	37.2	27.4	46.9	
Low	5.7	10.9	18.3	9.0	
Not recorded	2.7	3.1	6.5	3.5	
Flossing (%)					< 0.0001
Never	22.9	32.8	50.6	32.0	
Frequently	32.3	34.7	24.8	21.0	
Moderately	23.2	16.3	8.3	25.5	
Rarely	20.2	14.8	13.9	20.2	
Not recorded	1.3	1.4	2.4	1.3	
Arthritis (%)					< 0.0001
No	79.0	66.5	76.8	76.4	
Yes	20.8	33.1	22.9	23.3	
Not recorded	0.2	0.4	0.2	0.3	
Alcohol consumption (%)					< 0.0001
Mild	37.1	35.5	31.4	38.4	
Heavy	17.5	16.0	23.4	20.4	
Former	12.1	17.3	17.9	13.7	
Moderate	19.3	12.5	12.4	16.5	
Never	8.0	12.2	8.5	5.5	
Not recorded	6.1	6.5	6.5	5.6	
Hypertension (%)					< 0.0001
No	69.8	51.4	53.7	60.9	
Yes	30.2	48.6	46.3	39.1	
Smoking status (%)					< 0.0001
Never	64.8	48.9	36.5	55.6	
Former	23.7	30.6	27.5	24.9	
Current	11.5	20.5	36.0	19.5	
COPD (%)					< 0.0001
No	96.0	90.3	91.6	94.8	
Yes	4.0	9.7	8.4	5.2	
Cardiovascular disease (%)					< 0.0001
No	95.9	88.4	87.9	95.2	
Yes	4.1	11.6	12.1	4.8	
Diabetes (%)					< 0.0001
No	82.4	70.5	67.5	80.7	
Yes	17.6	29.5	32.5	19.3	
BMI (%)					0.0018
< 25	29.8	28.0	27.0	16.2	
≥25 - <30	34.3	35.0	37.6	38.3	
≥30	35.9	37.1	35.4	45.5	

Continuous variables: survey-weighted mean +/- SD, *P* value determined by survey-weighted linear regression

Categorical variables: survey-weighted percentage, *P* value determined by survey-weighted Chi-square tests

### The association between BMI and periodontitis

The association between BMI and periodontitis evaluated using regression analysis is presented in Table 2. In Adjusted Model II, the relationship between BMI (continuous or categorical variables) and periodontitis was not significant *(p* > 0.05). Moreover, after stratification by sex, CRP level and smoking status, a significant difference was not found (*p* > 0.05).

**Table 2:** Multifactorial regression analyses of BMI and periodontitis, stratified by sex, CRP level and smoking status

Exposure	Unadjusted model OR (95% CI) *P*	Adjusted Model I OR (95% CI) *P*	Adjusted Model II OR (95% CI) *P*
BMI	1.0 (1.0, 1.0) 0.279	1.0 (1.0, 1.0) 0.016	1.0 (1.0, 1.0) 0.532
BMI (categorical)			
BMI < 25 kg/m2	Ref	Ref	Ref
BMI ≥ 25 and < 30 kg/m2	1.1 (0.9, 1.3) 0.436	0.9 (0.8, 1.1) 0.406	0.8 (0.7, 1.0) 0.098
BMI ≥ 30 kg/m2	1.1 (0.9, 1.3) 0.191	1.1 (0.9, 1.4) 0.192	1.0 (0.8, 1.2) 0.680
*P* for trend	1.1 (1.0, 1.1) 0.195	1.1 (1.0, 1.2) 0.114	1.0 (0.9, 1.1) 0.856
Sex			
Male	1.0 (1.0, 1.0) 0.822	1.0 (1.0, 1.0) 0.432	1.0 (1.0, 1.0) 0.545
Female	1.0 (1.0, 1.0) 0.058	1.0 (1.0, 1.0) 0.015	1.0 (1.0, 1.0) 0.597
CRP level (tertile)			
≤ 0.91	1.0 (1.0, 1.1) 0.060	1.0 (1.0, 1.0) 0.793	1.0 (1.0, 1.0) 0.371
> 0.91, ≤ 1.9	1.0 (1.0, 1.0) 0.816	1.0 (1.0, 1.0) 0.831	1.0 (1.0, 1.0) 0.490
> 1.9	1.0 (1.0, 1.0) 0.226	1.0 (1.0, 1.0) 0.417	1.0 (1.0, 1.0) 0.201
Smoking status			
never	1.0 (1.0, 1.0) 0.290	1.0 (1.0, 1.0) 0.031	1.0 (1.0, 1.0) 0.347
former	1.0 (1.0, 1.0) 0.030	1.0 (1.0, 1.1) 0.006	1.0 (1.0, 1.0) 0.146
current	1.0 (1.0, 1.0) 0.360	1.0 (1.0, 1.0) 0.780	1.0 (0.9, 1.0) 0.153

Unadjusted model: not adjusted

Adjusted Model I: adjusted for age and sex

Adjusted Model II: adjusted for age, sex, race, marital status, annual family income level, alcohol consumption, hypertension, CRP, smoking status, COPD, cardiovascular disease, diabetes, flossing, and arthritis

### The association between CRP and periodontitis

The association between CRP and periodontitis examined using regression analysis is presented in Table 3. The relevant factors listed in Table 1 were modified in Adjusted Model II. However, in Adjusted Model II, for CRP as a continuous variable and CRP classified into three quartiles, both the *p* and *p* for trend values were higher than 0.05, indicating that the results were not statistically significant.

**Fig. 2:**
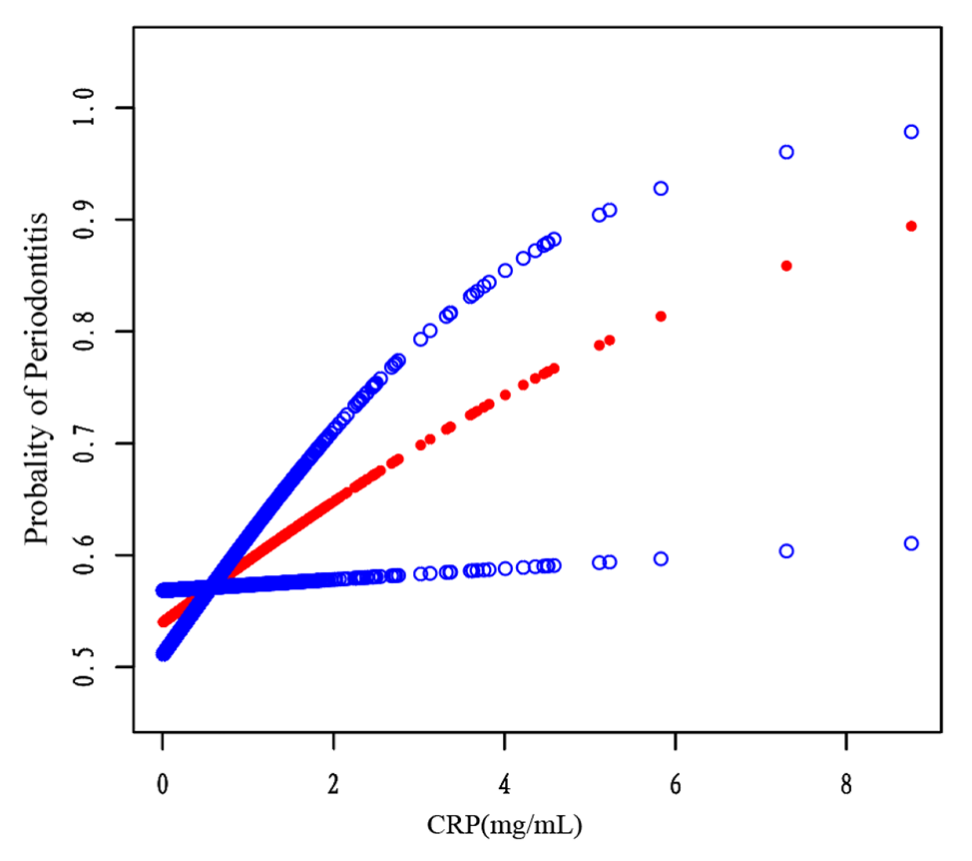
The association between CRP levels and periodontitis in a stratified population with a BMI ≥ 30 kg/m^2^ (red). The line formed by the blue circle indicates *the* 95% confidence interval (CI). The calculations were adjusted for the following parameters: age, sex, race, marital status, annual family income level, alcohol intake, hypertension, smoking status, COPD, cardiovascular disease, diabetes, arthritis, and dental flossing

Table 3 shows a statistically significant result with an OR of 1.2 (95% CI, 1.0 to 1.5) in Adjusted Model II for obese people with a BMI greater than 30 kg/m2 and a *p* value less than 0.05. This suggested that a 1-unit increase in the CRP was associated with a 20% increase in the relative risk for the development of periodontitis in the obese population. Increases in CRP are associated with an increased risk of periodontitis in those with a body mass index (BMI) of 30 kg/m^2^ or higher, as shown in Fig. 2.

**Table 3:** Multifactorial regression analyses of CRP levels and periodontitis, stratified by sex, BMI and smoking status

Exposure	Unadjusted model OR (95% CI) *p*	Adjusted Model I OR (95% CI) *p*	Adjusted Model II OR (95% CI) *p*
CRP	1.2 (1.1, 1.3) 0.003	1.3 (1.1, 1.4) < 0.001	1.1 (1.0, 1.3) 0.171
CRP (tertile)			
≤ 0.91	Ref	Ref	Ref
> 0.91, ≤ 1.9	1.2 (1.0, 1.4) 0.029	1.1 (1.0, 1.4) 0.118	1.0 (0.8, 1.3) 0.835
> 1.9	1.3 (1.1, 1.5) 0.003	1.4 (1.2, 1.7) < 0.001	1.1 (0.9, 1.4) 0.360
*p* for trend	0.003	< 0.001	0.362
Sex			
Male	1.4 (1.2, 1.8) 0.001	1.4 (1.1, 1.7) 0.006	1.1 (0.9, 1.4) 0.343
Female	1.2 (1.0, 1.4) 0.017	1.2 (1.0, 1.4) 0.018	1.1 (0.9, 1.3) 0.364
BMI			
BMI < 25 kg/m^2^	2.2 (1.4, 3.5) < 0.001	1.7 (1.1, 2.8) 0.031	1.3 (0.8, 2.0) 0.298
BMI ≥ 25 - <30 kg/m^2^	1.1 (0.9, 1.3) 0.314	1.1 (0.9, 1.3) 0.458	0.9 (0.8, 1.1) 0.397
BMI ≥ 30 kg/m^2^	1.1 (0.9, 1.3) 0.244	1.3 (1.1, 1.5) 0.005	1.2 (1.0, 1.5) 0.045*
Smoking status			
never	1.1 (1.0, 1.3) 0.145	1.2 (1.0, 1.4) 0.031	1.1 (0.9, 1.3) 0.479
former	1.2 (0.9, 1.5) 0.142	1.2 (0.9, 1.6) 0.124	1.0 (0.8, 1.3) 0.820
current	1.3 (1.0, 1.7) 0.082	1.3 (0.9, 1.8) 0.123	1.3 (0.9, 1.8) 0.207

* *p* < 0.05

Unadjusted model: not adjusted

Adjusted Model I: adjusted for age and sex

Adjusted Model II: adjusted for age, sex, race, marital status, annual family income level, BMI, alcohol consumption, hypertension, smoking status, COPD, cardiovascular disease, diabetes, flossing, and arthritis

We used a smoothed curve fit to confirm the results and found that the result of standard linear regression was still supported. In individuals with a BMI greater than 30 kg/m^2,^ the prevalence of periodontitis increased as CRP levels rose (Fig. 2).

## Discussion

After 2014, national health surveys rarely collected information on periodontal dental health, resulting in a scarcity of population-based data on the prevalence and risks of periodontitis in adults in the United States. We initially analysed periodontal data from 2009 to 2014. However, the lack of CRP data from 2011 to 2014 made data from 2009 to 2010 suitable for analysis.

This study sought to investigate the association between BMI and periodontitis and between CRP and periodontitis. An analysis of 3602 adults aged ≥ 30 years old was conducted in 2009–2010 using a nationally representative sample in the United States. Additionally, this study revealed an association between CRP levels and periodontitis in an obese population, independent of age, sex, race, marital status, annual family income level, alcohol consumption, hypertension, smoking status, COPD, cardiovascular disease, diabetes, flossing, and arthritis. This result indicates that the OR of periodontitis increases by 20% for a one-unit increase in the CRP level in the obese population.

Several recent studies have examined the association between BMI and periodontitis, reporting different results. Multivariate logistic regression analysis of data from the Fourth Korean National Health and Nutrition Examination Survey found no association between body mass index and periodontitis [21]. According to 224 university students, Ekuni D. et al. confirmed that increased BMI levels were linked to deteriorating periodontal disease [22]. BMI may have an inverse correlation with clinical AL, according to a Copenhagen study [12]. In a case‒control study with 79 participants, there was no significant difference in the prevalence of AL between the periodontal and control group of overweight individuals [23, 24]. In this study, which included a large sample of 3602 subjects, no association was found between BMI and periodontitis in analyses stratified according to obese severity. The difference among the results of the surveys might be due to the following reasons: (1) most previous clinical studies used a smaller sample size, whereas we used an NHANES sample representative of the US population; (2) the original study adjusted for fewer confounding factors, while the current experiment adjusted for more confounding factors, including age, sex, race, marital status, annual family income level, alcohol consumption, hypertension, smoking status, COPD, cardiovascular disease, diabetes, flossing, and arthritis; and (3) previous research applied the criterion of periodontal disease for investigation, which includes gingivitis and periodontitis, whereas this study used the CDC/AAP criteria, which refine the diagnosis of periodontitis and limit the illness’s scope.

Obesity is characterized by persistent inflammation [25, 26]. In addition to its function as a caloric reservoir, adipose tissue is a source of several proinflammatory cytokines, such as IL-6 [27, 28]. Inflammatory factor interleukin-6 (IL-6) is released into the bloodstream in response to inflammation, and this cytokine promotes the release of CRP [29], which is a pentraxin produced primarily by the liver and found in the bloodstream [30]. Alternatively, in patients with chronic periodontitis and chronic systemic diseases, higher levels of inflammatory cytokines are observed, including TNF-α, IL-1, and IL-6 [31]. In this study, we speculated on the relationship between CRP and periodontitis in the obese population. We propose two potential mechanisms that may explain this relationship: First, obesity is associated with inflammation [25, 26], which can stimulate the production of proinflammatory cytokines such as IL-6 [32]. IL-6, in turn, can stimulate and release more CRP [30], which can exacerbate the development of periodontitis by accumulating in the gingival crevicular fluid [33]. Second, as a chronic inflammatory condition, periodontitis can also secrete IL-6 and proinflammatory factors as from adipose tissue [28], which can increase CRP levels. The combined effect of these two inflammatory disease stimuli may lead to a more pronounced increase in CRP levels in individuals with both conditions. However, further studies are needed to confirm these hypotheses.

This cross-sectional study simply found an association between CRP levels and periodontitis in a population classified as obese by BMI, although the relationship between CRP levels and periodontitis has been validated in many studies [34]. In the chronic and aggressive periodontitis group, mean serum CRP and plasma fibrinogen levels were higher than those in the control group [35]. According to NHANES III data, a cross-sectional investigation of the US population revealed a link between periodontal disease and CRP [15]. These data were further corroborated by a large population-based investigation from Pomerania, demonstrating that both obesity and periodontitis are linked with increased systemic CRP and fibrinogen levels [36].

Notably, in a study with 46 participants, there was no significant change between the CRP levels of obese patients before and after periodontal therapy [37]. Another study of obese (n = 20) and normal-weight (n = 20) women with periodontitis discovered no correlation between CRP levels before and after periodontal treatment [38]. In addition, in a 4-week calorie intake intervention study including 53 young Koreans, CRP levels were not altered in the obese group [39]. In contrast, nonsurgical periodontal treatment has also been found to decrease serum levels of C-reactive protein [40]. We assume that the results of the irrelevance of pre- and post-periodontal treatment in an obese population may be attributable to the small sample size, population limitations, or the brief duration of periodontal treatment or monitoring of calorie control in individuals with obesity. The next step might be to consider a larger sample size for combined periodontal and obesity treatment, as well as a longer suitable observation time to monitor CRP levels and periodontitis.

Meisel et al. found that CRP may be a mediator of tooth loss in obese men [41]. They also found that periodontitis and obesity affected CRP in male subjects [42]. However, this study found no significant association for other individual groups. Nevertheless, we cannot ignore other conditions of periodontitis in specific obese groups, such as diabetes patients [43], menopausal women [44], and transgender people [45]. When treating periodontitis, it is important to consider a patient’s other medical conditions, ensuring that the treatment plan is tailored to their specific needs to achieve the best possible outcome for the their overall health.

Limitations.

There are three relevant flaws of our investigation. First, due to its cross-sectional design, this study was unable to establish a causal link between CRP levels and periodontitis in the obese group; therefore, we might choose to begin our next study from this point. Next, the NHANES database contains both CRP and periodontal examination results based on 2009–2010 data. We will continue to follow the NHANES updates and conduct pertinent analyses to validate the most recent available data, if applicable. Third, this study was unable to use the most up-to-date periodontitis classification criteria due to the unavailability of the required imaging data.

## Conclusion

Through data analysis, we found an association between CRP levels and periodontitis prevalence in the American population, although this association was only present in the obese population. While there are several hypotheses about the underlying mechanism, further studies are needed to validate these findings.

## Data Availability

The datasets generated and/or analysed during the current study are available in the National Health and Nutrition Examination Survey (NHANES) repository, which can be accessed at https://www.cdc.gov/nchs/nhanes/index.htm.
